# A Sweet Almond Globulin Multifunctional Peptide: Identification, In Silico Screening, Restraint Mechanisms to Keap1 and ACE, and Antihypertensive and Ferrous Transport Efficiency

**DOI:** 10.3390/nu17050907

**Published:** 2025-03-05

**Authors:** Bufan Xu, Peiyao Long, Yajun Zheng, Chen Feng, Yongliang Zhuang, Xinyi Wu, Siyin Zheng, Xinyu Liu, Yiheng Gao

**Affiliations:** 1 Food Science College, Shanxi Normal University, Taiyuan 030031, China; xubf98@163.com (B.X.); lpy166357173632023@163.com (P.L.); fengchen8098@163.com (C.F.); 17535861448@163.com (X.W.); 15977007452@163.com (S.Z.); 13935134643@163.com (X.L.); 18634382326@163.com (Y.G.); 2 School of Food Science and Engineering, Shaanxi University of Science and Technology, Xi’an 710021, China; 3 Yunnan Institute of Food Safety, Kunming University of Science and Technology, Kunming 650500, China; kmylzhuang@163.com

**Keywords:** multifunctional peptides, Kelch-like ECH-Associated Protein 1, angiotensin-I-converting enzyme, restraining mechanisms, spontaneous hypertensive rats, antioxidant, ferrous absorptivity, gastrointestinal stability

## Abstract

**Background:** Sweet almond expeller is an abundant protein resource, but there are few studies on multifunctional peptides. The purpose of this study is to improve its application in food and medical industries. **Methods:** This study investigated the identification, screening, and action mechanisms of antihypertensive peptides with antioxidant and ferrous binding activities derived from sweet almond globulin hydrolysates using intergrade in vitro and in silico methods and an animal model. **Results:** Eight novel oligopeptides were identified in sweet almond globulin hydrolysates subfraction D; of them, Pro-Met-Tyr-Gly-Gly-Gly-Met-Val (PMYGGGMV) exhibited ACE inhibitory activity (IC_50_: 121.16 μmol/L), ferrous binding ability (11.01 mg/g), and quenching capacities on hydroxyl (93.06%) and ABTS radicals (83.67%). The phenolic hydroxyl, amino, and carboxyl groups of PMYGGGMV were linked to Lys511, Tyr520, and Tyr523 in ACE’s substrate binding center through four short hydrogen bonds. PMYGGGMV can inhibit the Kelch-like ECH-Associated Protein 1 (Keap1)-nuclear factor erythroid 2-related factor 2 (Nrf2) interaction by binding to seven residues of Keap1 (including a key residue, Arg415). The ACE inhibitory and antioxidant activities of PMYGGMY were stable during gastrointestinal digestion. Ferrous chelation did not alter the ACE inhibitory and antihypertensive effects of PMYGGMY, but it reduced its ABTS and hydroxyl radical scavenging ability (*p* < 0.05). Additionally, PMYGGGMV reduced blood pressure of spontaneous hypertension rates and improved iron absorption across Caco-2 cells (*p* < 0.05). **Conclusions:** PMYGGGMV has the potential to prevent oxidative stress, hypertension, and iron deficiency.

## 1. Introduction

The cellular antioxidant system is an important defense mechanism to maintain bodily health, but excess reactive oxygen species can impair the function of this system, resulting in various chronic diseases, such as diabetes, hypertension, and cardiovascular diseases [1,2]. Oxidative reaction is an important factor in food deterioration, resulting in a significant decline in the nutritional value, color, taste, aroma, and texture of foods [3]. The Keap1-Nrf2-ARE system is critical for mitigating oxidative stress [4]. In this system, Kelch-like ECH-Associated Protein 1 (Keap1) negatively regulates nuclear factor erythroid 2-related factor 2 (Nrf2), which controls the expression of downstream antioxidant enzymes and cytoprotective genes. Moreover, the NRF2/KEAP1 system plays a key role in the onset and progression of several cancerous and non-cancerous diseases, such as periodontitis and cervical and endometrial cancers [5,6]. Peptides that can cause the separation of Keap1 and Nrf2 can promote the expression of cytoprotective genes and antioxidant enzymes and thus lower cellular oxidative pressure [7]. Recently, due to safety, economic factors, and availability, food-derived antioxidant peptides and their efficiency in food preservation and the prevention of cardiovascular diseases have received much attention [8,9]. Among cardiovascular diseases, hypertension is the dominant cause of cardiovascular diseases and causes a quarter of global mortality [10]. Although the specific mechanisms of hypertension remain unclear, the crucial role of angiotensin-I-converting enzyme (ACE) in controlling blood pressure has been clinically confirmed [11,12]. There are three key pockets (S1, S2, and S1′) in the substrate linking center and a zinc tetrahedron in the catalytic center of ACE [13]. ACE inhibitory peptides, especially those that can interact with the substrate binding center and the zinc tetrahedron of ACE, and antioxidant peptides that can relieve the oxidative pressure of vascular endothelial cells have potential antihypertensive effects [1,3]. Another feature of these ACE inhibitory peptides that can affect the zinc tetrahedron of ACE is that they have good metal ion chelation ability and iron fortification potential [11]. Furthermore, approximately 240 million people worldwide are threatened by iron deficiency anemia [10]. The predominant reasons for iron deficiency are the poor stability of iron in the gastrointestinal tract, low absorption rates, and the strong iron demand during pregnancy and infancy [13,14]. Acid–base changes, oxidative reactions, oxide, and nutrients in the digestive fluid of food, such as phytic acid, fiber, and metal ions, all convert food ferrous to trivalent iron, which cannot be absorbed by intestinal cells [15]. Compared with synthesized antioxidants, antihypertensive drugs, and common iron fortifiers (ferrous chloride and lactate), food-derived antioxidant and antihypertensive peptides and peptide–iron chelates are advantageous owing to their better safety, stability, scalability, and absorptivity [16,17,18]; however, their structure–activity relationship, specific action mechanisms, and functionalities in vivo have been scarcely studied [19]. Furthermore, increasing studies have studied the antioxidant, antihypertensive, or iron supplementary peptides from different food sources [20,21], but data on multifunctional peptides, especially food-derived peptides that simultaneously have antihypertensive, antioxidant, and iron supplementary activities, are scarce.

Sweet almond (*Runus amygdalus*) is used as an oil resource and as snake food ingredients. Its oil processing byproduct has a protein content of approximately 45 g/100 g and relatively balanced amino acid compositions [22]. Albumin and globulin account for 45.76 and 41 g/100 g of almond protein, respectively [23]. The functionalities of sweet almond albumin peptides, such as hypoglycaemia, antibacterial, antihypertensive, and anti-ultraviolet radiation activities, have been studied [21,24,25,26]. However, the functionalities of sweet almond globulin, especially its antioxidant, antihypertensive, and iron supplementary activities, remain unclear. Pre-experiments have shown that defatted sweet almond globulin hydrolysates (SAGHs) can effectively restrain ACE (64.32 ± 3.57%, at 1 mg/mL), scavenge ATBS and hydroxyl radicals (70.19% and 80.05%, respectively), and bind to ferrous ions (5.69 ± 0.11 mg/g). Herein, the objectives of this study were (1) purification, identification, silicon screening, and characterization of peptides with ACE inhibition, antioxidant, and ferrous binding capacities in SAGHs; (2) to investigate the mechanisms of action of the selected SAGH peptides on Keap1 and ACE; and (3) to study their antihypertensive effect on spontaneous hypertensive rats, their ferrous transport capacity, and their gastrointestinal stability. The relationship between antioxidant, hypotensive, and ferrous fortification activities of peptides will be further elucidated, and the study findings will offer novel strategies for the exploitation of foodborne multifunctional peptides.

## 2. Materials and Methods

### 2.1. Materials

Douspring Apricots Oil Processing Co., (Guangling, China) provided the sweet almond (*Runus amygdalus*) expeller powder that was produced on 12 July 2024. Pepsin (1:100,000 U·g^−1^, purity > 99.7%, Chemical Abstracts Service, CAS: 9001-75-6) and papain (8.0 × 10^5^ U/g, purity > 99.7%, CAS: 9001-73-4) were purchased from Nanjiang Enzymatic Reagent Co., Ltd. (Nanning, China). The Kunming Zoology Research Institute (Kunming, China) provided Caco-2 cells, fetal bovine serum (CAS: 11011-8611), and Hank’s Balanced Salt Solution (CAS: H4641). Captopril (purity > 96.1%, CAS: 62571-86-2), Dulbecco’s modified Eagle’s medium (CAS: R32206), Alcalase (from *Trichoderma Vride G*, 1.0 × 10^5^ U/g, purity > 99.7%, CAS: 9014-01-1), glutathione (CAS: 70-18-8), and trypsin (1:3000 U·g^−1^, purity > 99.7%, CAS: 9002-07-7) were obtained from Peisu Biotech. Co., Ltd. (Shanghai, China). ACE (0.1 U, from rabbit lungs, purity > 99.7%, CAS: 9015-82-1) was obtained from Sigma-Aldrich (St. Louis, MO, USA). All analytical-grade reagents, such as acetonitrile, β-D-deoxyribose (CAS: 533-67-5), and 2,2′-hydrazine-bis(3-ethylbenzothiazolin-6-sulfonic acid) diamine salt (ABTS, CAS: 30931-67-0), were obtained from Keoumi Chemicals Factory (Shanghai, China).

### 2.2. Extraction of Sweet Almond Expeller Globulin

Sweet almond expeller was dried at 45 ± 1 °C for 7 h using a 78HET-B blast drier (Shaoxing Drier Factory, Shaoxing, China) and then ground and sifted through a 60-mesh screen (DY-200, Dayong Vibration Equipment Co., Ltd., Xinxiang, China). The dried sweet almond expeller was defatted with petroleum ether II using the same procedures described by Zheng et al. [27]. Then, the deoiled sweet almond was thoroughly dispersed in 0.25 mol/L of NaCl (1:25, m/v). After 120 min of stirring at 175 r/min and 35 °C using the EDZF-C002 thermostatic vibrator, the mixture was filtered using a 113-25-Whatman paper, and the filtrate was centrifuged at 7300× *g*. The supernatant solution was poured into a dialysis bag with a cut-off weight of 7500 Da (Sanjiang Filtration Material Co., Ltd., Chengdu, China), sealed, and dialyzed against deionized water (dH_2_O) at 4 °C [28]. The dH_2_O was changed every 4 h. After 48 h, the dialysate was centrifuged (13,700× *g*, 15 min), and the pellet was lyophilized employing an EHI-220D lyophilizer (Lingling Lyophilize Instrument Co., Ltd., Wuchang, China) to obtain sweet almond expeller globulin. Before being lyophilized, the pellet was feezed at −40 °C for 2 h and then freeze-dried at 9.4 × 10^−4^ Pa for 8 h.

### 2.3. Hydrolysis of Sweet Almond Expeller Globulin

Sweet almond globulin was hydrolyzed using dual enzymes (Alcalase and papain) at the following conditions. First, 2 g/100 mL of defatted sweet almond globulin (100 mL) was hydrolyzed by 40 mg of Alcalase at 50 °C, 220 rpm, and pH 8.5 in a ZDF-C04 thermostatic vibrator for 90 min [28]. Second, papain (0.02 g) was added, and hydrolysis was continued at pH 7.5 and 50 °C, with a shaking rate of 220 rpm for 1 h. Afterwards, the proteolysis dispersion was heated at 100 °C for 10 min and centrifuged (13,700× *g*, 10 min). The supernatant was pooled and lyophilized using an EHI-220D lyophilizer to obtain SAGHs. The SAGHs’s degree of hydrolysis determination was conducted using the Nielsen method [29], whereas the protein content of SAGHs was measured following the Kjeldahl method [30].

### 2.4. Purification of SAGH Peptides

SAGHs (1.5 mg/mL) were filtrated on an ultra-membrane (diameter: 0.22 μm) (Jingfei Membrane Equipment Factory, Luzhou, China), and the filtrate (2 mL) was further isolated using column chromatography with Sephadex G-15 gel (Huxi Gel Chromatographic Column Factory, Shanghai, China) as the stationary phase. The gel was washed using deionized water (2.4 mL/min) for 300 min. The elution was collected using a BS-160A Automatic collector (Sanli Technology Co., Ltd., Shenzhen, China) and monitored at 280 nm [31]. The collected elution components were lyophilized using the EHI-220D lyophilizer and used to investigate the ACE inhibitory, ABTS radical (ABTS^+^) scavenging, and ferrous ion binding activities, respectively. ACE inhibitory activity was the first screening indicator, followed by ABTS+ scavenging activity. The amino acid sequences of the subfraction that had greater activity than other subfractions were analyzed.

### 2.5. Inhibitory Ability and Kinetics Towards ACE

The hippuric acid method [32] was used to measure the samples’ inhibitory ability towards ACE, following the same procedures as Zheng et al. [31]. The absorbance at 228 nm was the hippuric acid content and represented ACE activity; therefore, the inhibitory ability of samples towards ACE was defined as the percentage of reduction in the absorption at 228 nm between the control and sample groups. Moreover, the ACE inhibitory kinetic of SAGH peptides was investigated by employing a Lineweaver–Burk plot with *N*-hippuryl-l-histidyl-l-leucine (HHL, 0.13–1.32 mmol/L) as the substrate [33].

### 2.6. Antioxidant Activity

#### 2.6.1. ABTS^+^ Quenching Ability

The ABTS^+^ quenching ability was determined following the same procedures described by Vásquez et al. [1]. The same system without the sample was the control, and glutathione (0.1 mg/mL) was the comparison. The percentage of reduction in absorbance at 734 nm was defined as the ABTS^+^ scavenging ability of samples.

#### 2.6.2. Hydroxyl and Superoxide Radicals’ Scavenging Activity

As described by Wang et al. [34] and Vásquez et al. [1], the hydroxyl (·OH) and superoxide ($O_{2}^{-}\cdot$) radicals’ scavenging activity was measured using the β-deoxyribose oxidation and pyrogallol auto-oxidation methods, respectively. The comparison was conducted when the samples were replaced by glutathione (0.1 mg/mL).

#### 2.6.3. Reducing Power

The potassium ferricyanide method [35] was employed to determine the reducing power of the SAGH peptides at the same conditions reported by Wang et al. [34], where the absorbance at 700 nm represented the reducing power.

### 2.7. Ferrous Ion Binding Capacity

The ferrous ion binding capacity was determined using the *o*-phenanthroline method following the same procedures described by Xu et al. [28]. By plugging the absorbance value into the equation of *A* = 0.3719*C* + 0.0002 [36], the ferrous ion concentration (*C*) can be calculated. The ferrous ion binding capacity (mg/g) was defined as the decrease in ferrous concentration of the reaction solution per sample concentration.

### 2.8. Amino Acid Sequence Detection and Verification

The amino acid sequence was analyzed by following the same procedures as Li et al. [32] and using an LMS-6100B Hybrid-Triple-Quadrupole liquid–mass tandem mass spectrometry system (Agilent Technologies Inc., Santa Clara, CA, USA). The mode of the electrospray ionization needle was coupled G-1958 in terms of positive, and the analysis was conducted at a spray voltage of 4.4 kV, a spray flow rate of 50 μL/min, a data scanning range of 100–3000 *m*/*z*, an AGC target of 5 e^5^, a capillary temperature of 360 °C, and a mass resolution full width at half maximum of 70,000 [32]. PEAKS^®^ Studio 12.5 DeepNovo Peptidome software (Bioinformatics Solutions Inc., Waterloo, ON, Canada) was used to process the obtained mass spectrometry data. Peptide identification was accepted if it could be established with a probability > 80% [3]. National Center for Biotechnology Information (https://www.ncbi.nlm.nih.gov/guide/, accessed on 5 May 2024) was used for verification of the peptide sequences obtained.

### 2.9. In Silico Screening and Synthesis

Physicochemical properties and multifunctionalities of SAGH peptides, including antihypertension and antioxidant activity, were analyzed in silico with the AHTpin (https://webs.iiitd.edu.in/raghava/ahtpin/penta_design.php, accessed on 17 May 2024) and Peptide Ranker server (http://distilldeep.ucd.ie/PeptideRanker/, accessed on 17 May 2024) databases [37], respectively. When the response value predicted by Ranker was greater than 0.50 and the vector machine software score (SVMS) predicted by AHTpin was greater than 0.00, the peptide sequences were accepted as potential antioxidant and antihypertensive peptides [3]. The selected antioxidant and antihypertensive peptides were synthesized with a purity above 99.5% for the detection of antioxidant, ACE inhibition, and ferrous binding abilities.

### 2.10. Allergenicity and Toxicity Analysis

The sensitization and toxicity of SAGH peptides were analyzed in silico with the AlgPred (https://imtech.res.in/raghava/algpred/, accessed on 17 May 2024) and ToxinPred (https://imtech.res.in/raghava/toxinpred/, accessed on 17 May 2024) databases, respectively [37]. The threshold value for AlgPred prediction was 0.4 [38]. For toxicity prediction with ToxinPred, values of ‘−0.5’, ‘0’, and ‘+0.5’ meant non-toxic, unmatched, and toxic peptides, respectively [38].

### 2.11. Molecular Docking

TORSEP SLYBYII 2.0 software (SRULEXF-SCORK, Troesp Co., St. Louis, MO, USA) with a scoring function, Total-Score (T-score), Kuntz-D score, and consistency score (C-score) was used to determine the potential specific interaction modes of SAGH peptides with key antioxidant and antihypertensive targets in Keap1 (ID:2FLU, from https://www.rcsb.org/ (accessed on 22 May 2024) and ACE (PDB-108A, from https://rcsb.org/structure, accessed on 22 May 2024), respectively. Molecular docking conformations were acceptable when their T-score and C-score were at least 6.0 and 4.0, respectively [10].

### 2.12. Coordination Model Between SAGH Peptides and Ferrous Ions

First, the antioxidant and antihypertensive SAGH peptides and FeCl_2_ (0.13 mol/L) were reacted to prepare peptide–ferrous chelates with a mass ratio of 1:44 at pH 6.5, 175 rpm, and 30 °C for 30 min in an EDZF-C002 thermostatic oscillator [28]. Vitamin C (100 μmol/L) was added as a ferrous stabilizer. After centrifugation (6000× *g*, 25 min), the pellet was discarded, and the supernatant liquid was precipitated by four times the volume of anhydrous ethanol and lyophilized using a lyophilizer by employing an EHI-220D lyophilizer (Lingling Lyophilize Instrument Co., Ltd., Wuchang, China) to obtain the chelates. Afterwards, dry KBr (20 mg) was thoroughly mixed with the SAGH peptides (1 mg) or their ferrous chelates (1 mg) under an NL-3C Infrared baking lamp, respectively, and then pressed into 1–2 mm sheets [17]. Those sheets were analyzed with a Fourier transform infrared (FT-IR) spectrometer (LIDA-20, Hengchuanglida Precision Instrument Co., Ltd., Tianjin, China) at a wavenumber range of 4000–400 cm^−1^.

### 2.13. Gastrointestinal Stability of SAGH Peptides and Their Ferrous Chelates

As described by Wu et al. [39], the simulative gastric digestive fluid consisted of sodium chloride (0.18 mol/L), ultrapure water (180 mL), and 0.40 mg/mL of pepsin. The simulation intestinal digestive juice was composed of NaHCO_3_, (0.625 g/mL), bile salt (3 g/100 mL), pancreatin (0.35 mg/mL), and 180 mL of ultrapure water. The antihypertensive and antioxidant SAGH peptides (5 mg/mL, 60 mL) were first hydrolyzed using 150 mL of simulative gastric fluid at 37 °C and 140 rpm for 90 min. The mixture was adjusted to pH 7.0, and 180 mL of the simulative intestinal hydrolysis fluid was added and stirred at 37 °C for 2 h. After boiling for 6 min, the mixture was cooled with running water to room temperature. The ACE inhibition capacity and antioxidant ability of the treated SAGH peptides were detected and compared to those of the digested SAGH peptides.

The gastrointestinal stability of the ferrous chelate of SAGH peptides was studied as follows: 2.75 mg SAGH peptide–ferrous chelate was subjected to simulative gastric fluid hydrolysis at pH 1.9 ± 0.1, 175 rpm, and 37 °C using the EDZF-C002 thermostatic vibrator for 90 min [40]. Next, the intestinal digestive fluid was added, and digestion was continued at pH 6.8 for 2 h in the EDZF-C002 thermostatic vibrator (90 min, 175 rpm). During gastrointestinal digestion, ferrous gastrointestinal stability was represented by soluble ferrous content in the digestive dispersion, which was determined every 30 min following the *o*-phenanthroline method [36], and the sample data were compared with those of ferrous lactate and ferrous chloride (0.1 mg/mL).

### 2.14. Effects on Blood Pressures

Spontaneously hypertensive rats (SHRs, each weighting approximately 250 g, 14 weeks old) were obtained from Vitonglihua Test Animal Biotech. Co., Ltd. (Beijing, China) and used to investigate the hypotensive ability of ACE inhibitory and antioxidant SAGH peptides [41]. After 7 days of adaptation, the SHRs were assigned to the blank, positive, SAGH peptides, and SAGH peptide–ferrous chelate groups (each group contained six SHRs), which were orally administered NaCl (90 mg/mL), captopril (14 mg per body weight kg), peptides (50, 150, and 200 mg per body weight kg), and peptide–ferrous chelates (50, 150, and 200 mg per body weight kg) every day, respectively. At 0, 120, 360, and 600 min after oral administration, the systolic and diastolic blood pressures and heart rates of the SHRs were measured using a NIBP-M1 Rat-tail noninvasive blood pressure monitor (Yuyan Scientific Instrument Co., Ltd., Shanghai, China) following the tail cuff method [10].

### 2.15. Capacity to Promote Iron Transmembrane Absorption

In a 24-well Transwell culture plate, Caco-2 cells (0.75 × 10^6^ cells/cm^2^) were cultivated using Dulbecco’s modified Eagle’s medium that contained fetal bovine serum (20 g/L), streptomycin (1 μg/μL), penicillin (1 μg/μL), and neomycin (1 μg/μL), and it was refreshed every 48 h [16]. After 12–14 d of incubation at 5% CO_2_ and 37 °C, the transepithelial electrical resistance was more than 400 Ω·cm^2^, and a monolayer Caco-2 cell mode was formed. Afterwards, the medium was removed, and Hank’s Balanced Salt Solution was added. After 30 min of cultivation at 5% CO_2_ and 37 °C, Hank’s Balanced Salt Solution was removed from the apical side, and SAGH peptide–ferrous chelates (320 μg/mL) were added and then cultivated at 5% CO_2_ and 37 °C for 150 min. Every 30 min, the ferrous amount of the basolateral-side cultivate solution was measured using the *o*-phenanthroline method [36]. Ferrous lactate and FeCl_2_ (0.3 mg/mL) were subjected to the same treatment for comparison.

### 2.16. Statistical Analysis

Tests were conducted at least three times. The data were analyzed using V.17.4 SPSS (IBM Company, Armonk, NY, USA) software. Data were expressed as mean ± standard error. Statistical analysis was performed through one-way analysis of variance (ANOVA), and the significant difference was analyzed using Duncan’s multiple comparisons. *p* < 0.05 indicated a statistically significant difference.

## 3. Results

### 3.1. Separation of Peptides According to ACE Inhibitory and Antioxidant Activity

**Figure 1:**
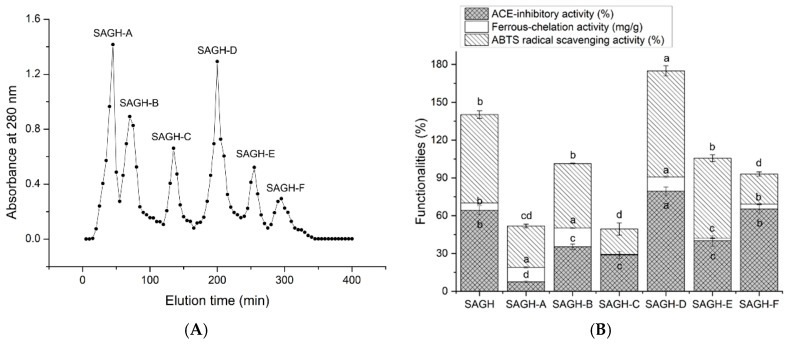
(**A**) The six subfractions (SAGH-A, SAGH-B, SAGH-C, SAGH-D, SAGH-E, and SAGH-F) separated from sweet almond globulin hydrolysates (SAGHs) after Sephadex G-15 gel chromatography and (**B**) their ability to inhibit ACE, quench ABTS radicals, and chelate ferrous ions. Lowercase letters (a–d) above the bars represent significant differences in the same type of functionality (*p* < 0.05). Tests were conducted in triplicate (*N* = 3).

The hydrolysis degree of SAGHs by Alcalase and papain was 40.07 ± 6.52%. The ABTS^+^ scavenging activity of SAGHs, and their ability to inhibit ACE and ferrous chelation, were 70.19 ± 2.96%, 64.32 ± 3.57%, and 5.69 ± 0.11 mg/g, respectively. The guanidine, imidazolyl, and phenolic hydroxyl groups in peptides can quickly supply protons to quench free radicals [42]. Following Sephadex G-15 gel chromatographic separation, six fractions (SAGH-A, SAGH-B, SAGH-C, SAGH-D, SAGH-E, and SAGH-F) were isolated from SAGHs (Figure 1A). As shown in Figure 1B, the SAGH-D showed the highest ability to quench ABTS+ and inhibit ACE (*p* < 0.05). Moreover, SAGH-D showed considerable ferrous chelating ability (11.40 ± 0.64 mg/g). Therefore, SAGH-D’s amino acid sequence was further identified with ESI-MS/MS.

### 3.2. Identification, In Silico Screening, and Structure–Activity Relationship Analysis

**Figure 2:**
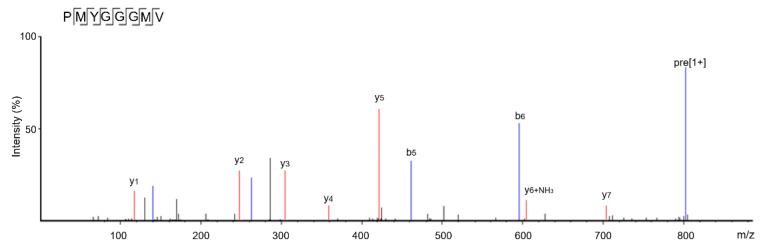
The second electrospray tandem mass spectra of the peptide PMYGGGMV identified in sweet almond globulin hydrolysates. Tests were conducted in triplicate (*N* = 3).

**Figure 3:**
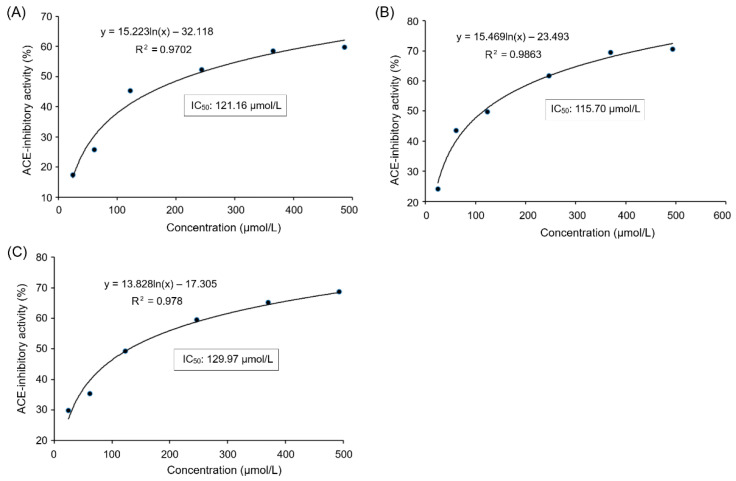
The regression analysis of ACE inhibitory activities of PMYGGGMV (**A**), PMYGGGMV-ferrous chelate (**B**), and PMYGGGMV (**C**) after simulated gastrointestinal digestion. IC_50_ means the amount of the samples required to inhibit half of the activity of ACE. Tests were conducted in triplicate (*N* = 3).

The peptides with greater than 12 amino acid residues were generally not selected because of their sensitivity to digestive enzymes, low absorption rate, and potential sensitization [20]. The identification results show that there were eight oligopeptides in SAGH-D: Ala-Glu-Ala-Pro-Lys-Glu (AEAPKE, 643.76 Da), Arg-Ser-Gly-Gly-Glu-Glu-Ala (RSGGEEA, 704.78 Da), Pro-Met-Tyr-Gly-Gly-Gly-Met-Val (PMYGGGMV, 811.10 Da), Asn-Asp-Ser-Ala-Gly-Ser (NDSAGS, 549.56 Da), Arg-Phe-Met-Thr-Tyr-Ser (RFMTYS, 804.00 Da), Arg-Phe-Met-Thr-Tyr-Ser-Ser-Ser (RFMTYSSS, 775.87 Da), Arg-Ser-Gly-Gly-Glu-Glu-Ala-Ala (RSGGEEAA, 775.87 Da), and Ser-Gly-Gly-Glu-Glu-Ala-Ala-Ala-Gly-Pro (SGGEEAAAGP, 1001.16 Da) (Table 1). Of them, the prediction with the database AHTpin and the Peptide Ranker server revealed that PMYGGGMV was an antihypertensive and antioxidant sequence given its vector machine software scores and response values of 0.77 and 0.56, respectively [19]. Figure 2 shows the electrospray tandem mass spectra of PMYGGGMV. As shown in Figure 3A, ACE inhibition by PMYGGGMV showed a logarithmic response, with a relatively low IC_50_ value of 121.16 μmol/L. Moreover, the results in Table 2 show that the chemically synthesized PMYGGGMV had high antioxidant activity, including reducing power (0.484), and quenching abilities on hydroxyl (97.49%), ABTS (92.55%), and superoxide radicals (73.76%) at 100 μg/mL. As AEAPKE, RSGGEEA, NDSAGS, RFMTYS, RFMTYSSS, RSGGEEAA, and SGGEEAAAGP did not show any potential antihypertension (vector machine software scores < 0, Table 1), their ACE inhibitory activities were not detected.

### 3.3. Ferrous Chelating Ability

As shown in Table 1, the oligopeptides identified in SAGH-D (AEAPKE, RSGGEEA, PMYGGGMV, NDSAGS, RFMTYS, RFMTYSSS, RSGGEEAA, and SGGEEAAAGP) offered abilities to chelate ferrous ions; of them, RSGGEEA showed the highest ferrous binding power (22.65 mg/g), followed by AEAPKE and SGGEEAAAGP (16.14 and 18.22 mg/g, respectively). PMYGGGMV showed a considerable ability to chelate ferrous ions (11.01 mg/g), too. The Met and Gly in PMYGGGMV can effectively bind to ferrous ions [13,17]. Ascribed to its excellent metal ion chelating capacity, Gly is an ideal ingredient for iron or calcium supplements [43]. Additionally, the carbonyl group and amido bond in PMYGGGMV showed ferrous ion binding ability [28]. As RSGGEEA, AEAPKE, and SGGEEAAAGP did not show any potential antihypertensive or antioxidant activity, they were not selected in this study.

### 3.4. Physicochemical Characteristics

Prior studies have shown that peptides with high hydrophobicity can effectively prevent oxidation and hypertension by binding to Keap1 and ACE, respectively [10,28]. High hydrophilicity indicates that peptides have relatively strong affinity for metal ions [39]. Furthermore, the amphiphilicity represents the ratio of hydrophilicity to hydrophobicity [3]. As shown in Table 1, the hydrophobicity and amphiphilicity of PMYGGGMV were 0.19 and 0.63, respectively, corresponding to its high antioxidant, ACE inhibitory, and ferrous binding activities (Table 1 and Figure 3A). Additionally, ferrous fortification of PMYGGGMV may be reduced at its isoelectric point of 5.88 because of the lower surface charge [13].

### 3.5. Virtual Analysis of Security

Sensitization and toxicity must first be considered when peptides are applied to food. As shown in Table 1, AEAPKE, RSGGEEA, PMYGGGMV, NDSAGS, RFMTYS, RFMTYSSS, RSGGEEAA, and SGGEEAAAGP did not show any potential allergenicity, as their allergenic prediction results were negative (Table 1). Moreover, their toxic prediction values were −0.5 (Table 1), indicating that they were non-toxic peptides [38]; however, toxicological tests should be performed in further work.

### 3.6. Inhibition Mechanisms Towards ACE and Keap1

#### 3.6.1. Molecular Docking of ACE with SAGH Peptides

**Figure 4:**
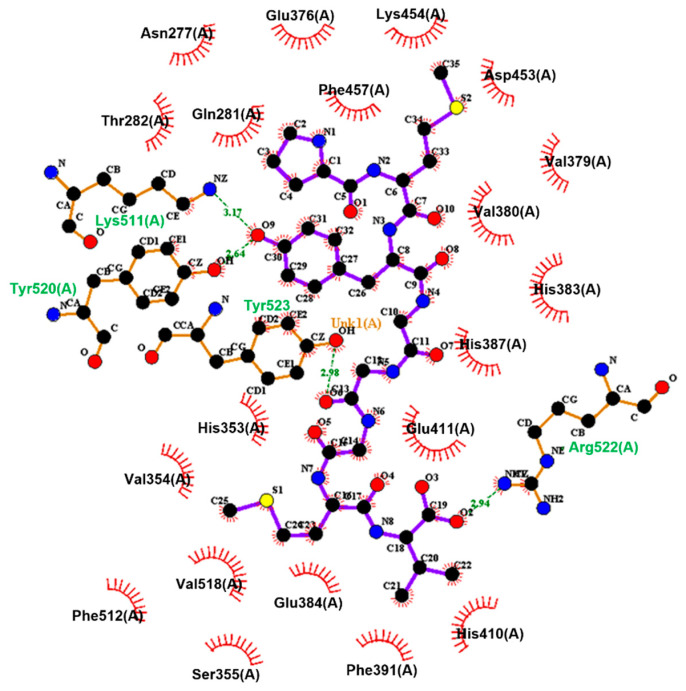
The binding details of ACE (PDB: 1O8A) with PMYGGGMV. The black, red, yellow, and blue balls represent carbon, oxygen, sulphur, and nitrogen atoms, respectively. Purple lines are representative of peptides, and red eyelashes indicate the hydrophobic interactions between PMYGGGMV and ACE. The green dotted line and the numbers on it indicate the hydrogen bond and the distance of the hydrogen bond, respectively. Tests were conducted in triplicate (*N* = 3).

The molecular docking results (Figure 4) revealed that the Tyr residue’s phenolic hydroxyl group in PMYGGGMV was linked to Lys511 and Tyr520 (belonging to S2) in ACE via two short hydrogen bonds. The carbonyl group of the fifth Gly residue in PMYGGGMV can bind to the phenolic hydroxyl group of Tyr523 in ACE, which is located in the substrate binding center S1, whereas the carboxyl group of Val in the *C*-terminal of PMYGGGMV formed a hydrogen bond with the imidazolyl group of Arg522 in ACE. Moreover, hydrophobic interactions were observed between PMYGGGMV and twenty residues of ACE (Table 3), including Glu411, His383, and His387, which are key residues in the zinc tetrahedron [32]. Thus, PMYGGGMV can restrain ACE by affecting the substrate binding sites of ACE or by impacting its zinc tetrahedron. Additionally, the distance of hydrogen bonds between PMYGGGMV and ACE was short (2.64–3.17 Å), and the T-scores for the domain docking models of ACE PMYGGGMV (11.86, Table 3) were much higher than the threshold (6.0) [19], corresponding to its high ACE inhibitory activity.

#### 3.6.2. Molecular Docking of Keap1 with SAGH Peptides

**Figure 5:**
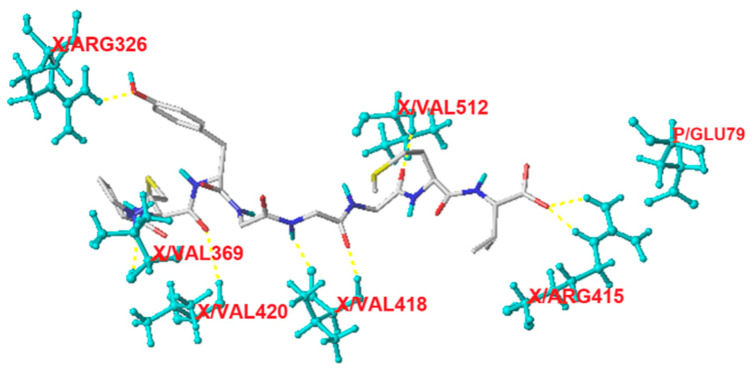
The interaction models for hydrogen bonding interactions within Keap1 (PDB ID: 2FLU) and PMYGGGMV. The tests were conducted in triplicate (*N* = 3).

The Keap1-Nrf2-ARE system is crucial for mitigating oxidative damage, in which Keap1 links with Nrf2 and regulates its transcription [4]. Antioxidants can inhibit the interaction between Nrf2 and Keap1 and increase free Nrf2, which is conducive for the subsequent expression of cytoprotective genes and antioxidant enzymes, thereby lowering oxidative pressure [32]. As shown in Figure 5 and Table 3, eight short hydrogen bonds (2.12–3.19 Å) are found between PMYGGGMV and eight residues in Keap1 (Arg326, Val369, Val512, Val420, Val418, Arg415, and Glu79), suggesting that PMYGGGMV has relatively strong affinity with the Kelch domain of Keap1 and consequently inhibits the interaction between Nrf2 and Keap1 [3].

#### 3.6.3. Restraint Kinetics

**Figure 6:**
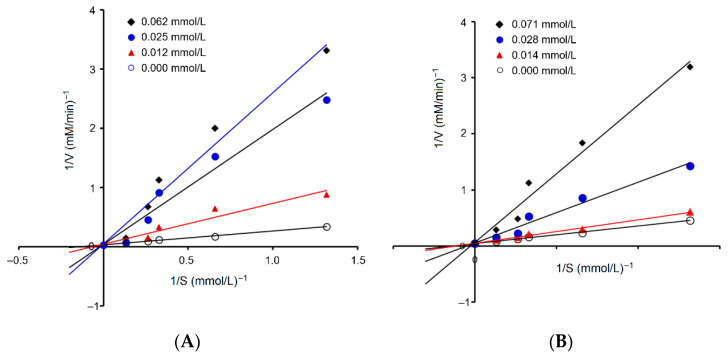
Lineweaver–Burk plots of ACE inhibition of PMYGGGMV (**A**) and PMYGGGMV–ferrous chelate (**B**). The tests were conducted in triplicate (*N* = 3).

Figure 6A depicts the effects of the addition of PMYGGGMV on the production rate of hippuric acids by ACE hydrolysis of HHL. Based on the Michaelis–Menten kinetic curves with an increasing dose of PMYGGGMV, the maximum velocity (*V*_max_) was not altered, but the *K_m_* value increased, verifying that the addition of PMYGGGMV lowered the production rate of hippuric acids by competitively binding to the binding center of the substrate in ACE and restraining its affinity to the substrate (HHL). Correspondingly, Figure 4 shows that PMYGGGMV formed short hydrogen bonds with active residues in ACE’s active centers, S1 and S2.

### 3.7. Chelation Patterns Between Ferrous Ions and PMYGGGMV

**Figure 7:**
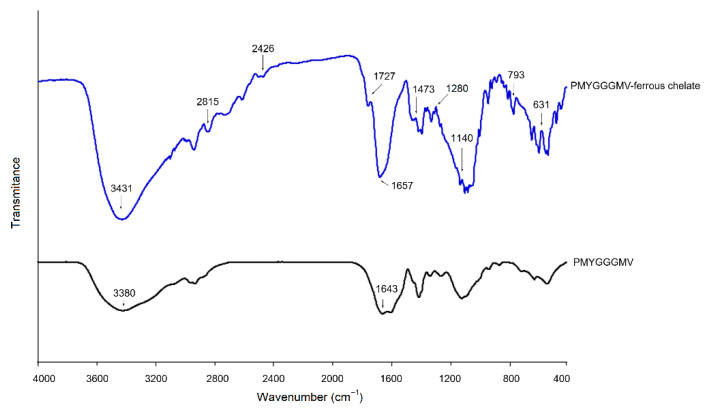
Fourier-transformed infrared spectra of PMYGGGMV and PMYGGGMV–ferrous chelate. The tests were conducted in triplicate (*N* = 3).

The FT-IR spectra in Figure 7 depict the ferrous chelation patterns of PMYGGGMV. Slight differences can be seen between the FT-IR spectra of PMYGGGMV–ferrous chelate and PMYGGGMV. After ferrous chelation, the branded peak at 3380 cm^−1^ in the spectrum of PMYGGGMV shifted to 3431 cm^−1^, which is ascribed to the interactions between ferrous ions and their hydroxyl groups [43]. The blue shift (from 1643 to 1657 cm^−1^) that appeared in the spectrum of PMYGGGMV after ferrous chelation confirmed the binding force of the carbonyl groups of the amide band I to ferrous ions [18]. Moreover, the carbon–nitrogen bond in amide band III of PMYGGGMV chelated ferrous ions because new peaks appeared at 1473 cm^−1^ in the spectrum of PMYGGGMV–ferrous chelate [39]. The affinity of the phenolic hydroxyl group of the Tyr residue in PMYGGGMV to ferrous ions was verified by the new peak that appeared at 1280 cm^−1^ (representative of the aromatic acids and methyl groups in the benzene ring) in the spectrum of PMYGGGMV–ferrous chelate [15]. Additionally, a new peak was found at 1727 cm^−1^ after chelation of PMYGGGMV, confirming the linkage of sulfur atoms of Met with ferrous ions [11]. Thus, ferrous ions were chelated by the carboxyl, methylmercapto-, phenolic hydroxyl, and amido groups in PMYGGGMV. A similar trend was observed by Chen et al. [14]. However, further studies are required to investigate more specific coordination modes.

### 3.8. Effect of Ferrous Chelation on Antioxidant and ACE Inhibition Activities of PMYGGGMV

As shown in Figure 3B, PMYGGGMV–ferrous chelate exhibited an ACE inhibitory IC_50_ value of 115.70 μmol/L, which was not significantly different from that of PMYGGGMV (121.16, Figure 3A), highlighting that the ACE inhibition capacity of PMYGGGMV was not notably altered by ferrous chelation. Moreover, as shown in Figure 6B, the addition of PMYGGGMV–ferrous chelate increased the *K_m_* value of the Michaelis–Menten kinetic curves but did not change the *V*_max_, verifying that PMYGGGMV was a competitive ACE inhibitor [3]. Thus, the ACE inhibitory activity and model of PMYGGGMV were not changed by ferrous chelation because of the strong affinity of PMYGGGMV for ACE [44].

As shown in Table 2, the ABTS and hydroxyl radical scavenging activity and the reducing power of PMYGGGMV–ferrous chelates were lower than those of PMYGGGMV (*p* < 0.05), even though PMYGGGMV–ferrous chelates exhibited considerable antioxidant activity, including reducing power (0.179) and quenching abilities on hydroxyl (60.04%) and superoxide radicals (75.49%, Table 2). Moreover, the high ferrous chelation ability (10.67 mg/g, Table 1) suggested that PMYGGGMV can take away the catalyst of the oxidation reaction and thus inhibit free radicals’ chain oxidation [43].

### 3.9. Gastrointestinal Stability

#### 3.9.1. Gastrointestinal Stability of PMYGGGMV

Good gastrointestinal stability is one of the prerequisites for peptides to exert antihypertensive and antioxidant activities in vivo [45]. As shown in Figure 3C, the gastrointestinally hydrolyzed PMYGGGMV exhibited considerable ACE inhibition ability (IC_50_: 129.97 μmol/L) and antioxidant activity (Table 2), which were not different from those of the digested PMYGGGMV (Figure 3A and Table 2) (*p* > 0.05), suggesting that the ACE inhibition and antioxidant activities of PMYGGGMV were stable during gastrointestinal digestion.

#### 3.9.2. Gastrointestinal Stability of the PMYGGGMV–Ferrous Chelates

**Figure 8:**
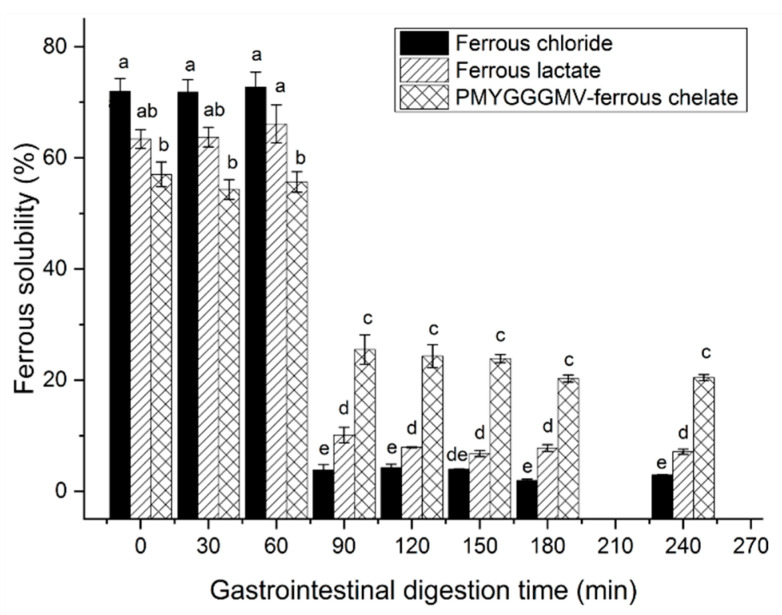
Ferrous solubility of the ferrous chloride, ferrous sulfate, and PMYGGGMV–ferrous chelate against simulated gastrointestinal digestion. Different lowercase letters (a–e) on the bars are representative of significant differences (*p* < 0.05). The tests were conducted in triplicate (*N* = 3).

During gastric digestion (0–90 min), the ferrous solubility of ferrous lactate, ferrous chloride, and PMYGGGMV–ferrous chelate was relatively stable (Figure 8) because ferrous ions are stable at acidic conditions [17]; however, their ferrous solubility apparently decreased at 91–240 min (*p* < 0.05) because soluble ferrous ions were perhaps converted to insoluble compounds when the pH value increased to 7.0 [43]. The same results were found in prior reports [28,39]. More importantly, from 90 to 150 min, the ferrous solubility of PMYGGGMV ferrous chelates was much higher than those of ferrous lactate and ferrous chloride, indicating that PMYGGGMV is better at improving ferrous gastrointestinal stability (*p* < 0.05). One reason for the high ferrous solubility of the PMYGGGMV–ferrous chelate was its excellent ferrous binding ability, hydrophilicity (11.01, Table 1), and gastrointestinal stability (Figure 3C). Peptides with greater stability and hydrophilicity are better at improving iron’s gastrointestinal stability [16]. However, more studies are needed to investigate the special effect of gastrointestinal hydrolysis on the interactions between ferrous ions and PMYGGGMV.

### 3.10. Hypotensive Effect

**Figure 9:**
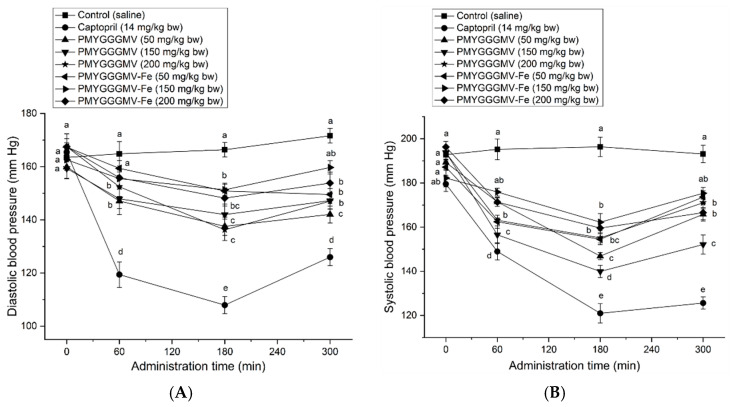
(**A**) Influence of oral administration with PMYGGGMV and PMYGGGMV–ferrous chelate on diastolic blood pressure (**A**) and systolic blood pressure (**B**) on spontaneous hypertensive rats (SHRs). The SHRs in the sample groups were orally administered peptides or peptide–ferrous chelate at 50, 150, and 200 mg/kg/body weight every day. The SHRs of the positive control group were given captopril at 14 mg/kg/body weight once daily, whereas the SHRs in the control group were just given 0.5 mL of NaCl (0.9 g/100 mL). Small letters on the data points (a–e) mean significant differences (*p* < 0.05). The tests were conducted in triplicate (*N* = 3).

As shown in Figure 9A,B, from 1 h of dosing at 50–200 mg/kg body weight, PMYGGGMV and PMYGGGMV–ferrous chelate apparently lowered the diastolic and systolic blood pressures of spontaneous hypertension rats (*p* < 0.05), predominately due to their considerable ACE inhibitory activity (Table 1 and Figure 3) and relatively good gastrointestinal stability (Figure 8) [9]. Prior studies have found that peptides can exhibit antihypertensive effects in vivo if they have good ACE inhibitory abilities, gastrointestinal stability, and absorption, too [36,41]. Moreover, an increase in the dose did not improve the blood pressure lowering effect of PMYGGGMV–ferrous chelate and PMYGGGMV (*p* > 0.05), revealing that PMYGGGMV–ferrous chelate and PMYGGGMV were not dose-dependent antihypertensive peptides. Furthermore, the hypotensive effect of PMYGGGMV–ferrous chelate was not different from that of PMYGGGMV (*p* > 0.05) regardless of diastolic or systolic blood pressure, confirming that ferrous chelation had no notable effect on the hypotensive activity of PMYGGGMV, as ferrous chelation did not alter the ACE inhibition capacity or model of PMYGGGMV (Figure 3A,C and Figure 6A,B).

### 3.11. Transmembrane Absorption of Ferrous Ions

**Figure 10:**
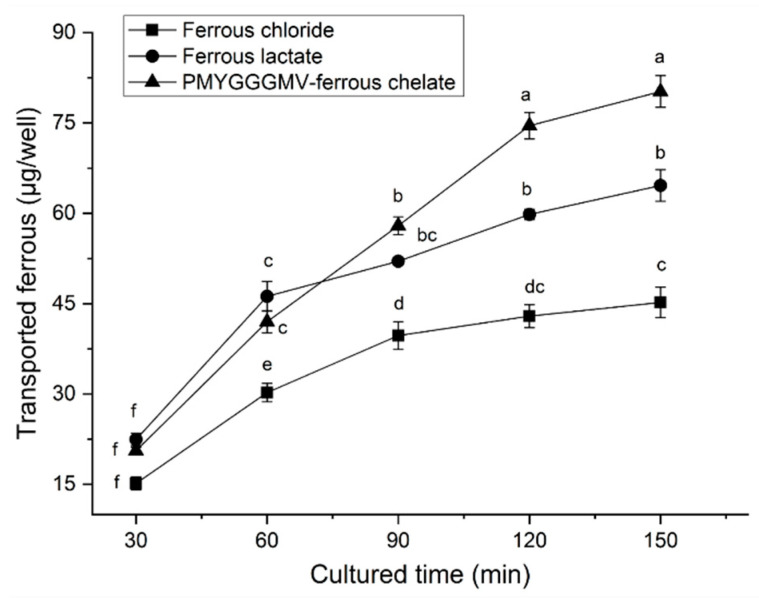
The amount of ferrous ions transported across Caco-2 cell monolayers by ferrous lactate, FeCl_2_, and PMYGGGMV–ferrous chelate. The tests were conducted in triplicate (*N* = 3). Different smaller letters (a–f) on the data points mean significant difference (*p* < 0.05).

The ability of PMYGGGMV–ferrous chelate to improve ferrous transmembrane absorption was greater than that of ferrous chloride (*p* < 0.05, Figure 10). During 120–150 min, PMYGGGMV–ferrous chelate was better (*p* < 0.05) at transporting iron across the monolayer of Caco-2 cells than ferrous lactate (which is widely used as an adjunct to the treatment of anemia) [17], suggesting that PMYGGGMV–ferrous chelate has potential as an iron supplement.

## 4. Discussion

To obtain multifunctional peptides from AGH, Alcalase and papain were used to hydrolyze sweet almond globulin in this study. The SAGHs showed excellent ACE inhibitory and antioxidant activity, and they showed a higher degree of hydrolysis than that of almond protein hydrolyzed with Alcalase [25]. The guanidine, imidazolyl, and phenolic hydroxyl groups in peptides can quickly supply protons to quench free radicals [42]. The γ-carboxyl and ε-amino groups in polar amino acids of peptides, such as Asp, His, and Glu, can easily bind to ferrous ions [13], whereas hydrophobic amino acid residues have been found to remarkably restrain ACE [31]. Papain and Alcalase preferentially hydrolyze peptide sequences rich in hydrophobic (Leu, Phe, Ile, Val, Met, and Ala) and polar amino acid residues [33,43]; therefore, they were used to prepare SAGH antioxidant and antihypertensive peptides in this study.

PMYGGGMV showed a low IC_50_ value of 121.16 μmol/L (Figure 3A). As a decrease in the IC_50_ value indicates an increase in the ACE inhibition capacity of peptides [32], PMYGGGMV showed better ACE inhibitory ability than peptides of GCHHY from millet bran glutelin-2 (IC_50_: 147 μmol/L) [29] and VIPTEPPHA from Faba beans (IC_50_: 259.7 μmol/L) [43] but a lower ability than Captopril (IC_50_: 0.14 μmol/L), which is widely used for antihypertension [10]. Prior studies have confirmed that a peptide will be better at inhibiting ACE if its *C*-terminal tripeptide contains Phe, Tyr, Arg, or Pro [46]. Special amino acid residues in the *N*-terminal, such as Ser, Tyr, Val, Gln, and Pro, can notably improve peptides’ restraining capacity on ACE [9]. Now, in silico techniques have revealed that the guanidyl, sulfhydryl, γ-hydroxyl, or ε-amino groups of the peptides can bind to key residues of ACE [19,33,47]. Therefore, the Pro, Tyr, Met, and Val residues in PMYGGGMV predominately contributed to its inhibition ability towards ACE.

Alternatively, the guanidine of Arg, imidazolyl in His, the free hydroxyl group of Ser, the cyclic amino group of Pro, and the phenolic hydroxyl group of Tyr can quickly quench free radicals, showing excellent antioxidant ability [42]. Moreover, the sulfhydryl group in Cys or Met can inhibit free radicals’ chain reaction by chelating metal ions, which are catalysts for oxidation reactions [46]. Therefore, the residues, including Tyr, Pro, Val, and Met, were responsible for the antioxidant activity of PMYGGGMV. Furthermore, the repeated amino acid sequences “GGG” can enhance the antioxidant activity of PMYGGGMV [7]. Peptides containing Ser, Tyr, Val, or Pro, such as VSRRFIYYL and SPAIPLP from broad bean [3] and YLSF and LPSYVN from apricot [21], all exhibit excellent antioxidant activity.

The molecular docking results in Figure 4 show that PMYGGGMV can link with Lys511, Tyr520, and Tyr523 in ACE’s substrate binding center through four short hydrogen bonds. Prior studies found that peptides that can affect the substrate binding center (S1, S2, and S1’ pockets) or the catalytic triad (containing a zinc tetrahedron) of ACE were better at inhibiting ACE [48]. The results in Figure 6 confirm that PMYGGGMV was a competitive inhibitor of ACE. Peptides with a competitive inhibition model are better at inhibiting ACE [49]. Moreover, PMYGGGMV can inhibit the Kelch-like ECH-Associated Protein 1 (Keap1)-nuclear factor erythroid 2-related factor 2 (Nrf2) interaction by binding to seven residues of Keap1. Among these resides, Arg415 has been proven to be a key residue through which Keap1 binds to Nrf2 [7]. Prior studies have confirmed that binding to Arg415 can apparently lower the inhibiting ability of Keap1 on the transcription of Nrf2 and thus mitigate cellular oxidative damage [3,49]. Thus, the findings in this study indicate that PMYGGGMV can effectively inhibit ACE and weaken Keap1–Nrf2 interaction. However, specific interactions between PMYGGGMV and ACE or Keap1 require more research.

The results in Figure 3B and Figure 6B revealed that the ACE inhibitory activity and model of PMYGGGMV were not changed by ferrous chelation because of the strong affinity of PMYGGGMV for ACE [44]. As PMYGGGMV can form short hydrogen bonds with three active sites (Lys511, Tyr523, and Tyr520) in ACE’s substrate binding center (Table 3 and Figure 4), it is impossible for ferrous chelation to completely restrain the interactions between these key residues and PMYGGGMV [43]. Xu et al. [28] found that zinc chelation did not alter the ACE restraining model of millet peptides. Alternatively, ferrous chelation decreased the antioxidant activity of PMYGGGMV, including ABTS and hydroxyl radicals’ scavenging activity and reducing power (Table 2), probably because ferrous chelation restrained the ability of PMYGGGMV to absorb electrons or give protons [7], resulting in lower oxidation resistance. However, the specific influence of ferrous chelation on the structure and electrochemical properties of PMYGGGMV require further study.

The ACE inhibition and antioxidant activities of PMYGGGMV were stable during gastrointestinal digestion (Figure 3A and Table 2), which is mainly ascribed to the Pro, Val, and Met residues. Pro residue has been proven to notably increase peptides’ gastrointestinal stability because of its rigid amino ring [47,50], and branched amino acids (Val, Ile, and Leu) can increase the steric hindrance of peptides, which is conducive to the peptides’ stability [39]. Therefore, the Pro and Val residues of PMYGGGMV were mainly responsible for its gastrointestinal stability. Peptides rich in Pro and branched amino acid residues, such as VIPTEPPHA from Faba beans [43] and PIIAKMY from millet glutelin-2 [28], showed good gastrointestinal stability. However, more research is needed to investigate the special effect of gastrointestinal hydrolysis on the structure of PMYGGGMV.

The main reasons why PMYGGGMV–ferrous chelate showed a greater ability to transport iron across the monolayer of Caco-2 cells (Figure 10) were its greater ferrous chelating ability (Table 1) and gastrointestinal stability (Figure 8). Singh and Vi [40] and Ding et al. [13] found that ferrous ions chelated with peptides can avoid oxidation and thus have a higher transport rate. Furthermore, ferrous ions are mainly absorbed through the ion channel pathway (containing multiple carriers and enzymes) [16], while binding to PMYGGGMV may alter the in vivo absorption of ferrous ions. Prior studies have found that transporter (PepT1), interstitial cell, and endocytosis channels are the main absorption mechanisms of peptide–ferrous chelate, which are faster and require less energy consumption [32,39]. However, more special in vivo absorption mechanisms of PMYGGGMV iron chelate require further study.

## 5. Conclusions

A novel and safe multifunctional peptide, PMYGGGMV, was obtained from sweet almond globulin hydrolysates by combining in vitro and in silico methods. PMYGGGMV showed high ACE inhibitory activity (IC_50_: 121.16 μmol/L), reducing power, and quenching capacities on hydroxyl (97.49%), ABTS (92.55%), and superoxide radicals (73.76%) and ferrous binding ability (11.01 mg/g). The phenolic hydroxyl, amino, carboxyl, and γ-hydroxyl groups of PMYGGGMV competitively bound to ACE’s substrate binding centers, S1 and S2, or the zinc tetrahedron through short hydrogen bonds or hydrophobic interactions. PMYGGGMV can inhibit Keap1–Nrf2 interaction by binding to seven residues of Keap1 (including the key residue Arg415). Ferrous ions were mainly chelated by the carboxyl, phenolic hydroxyl, and amide groups of PMYGGGMV. PMYGGGMV exhibited notable hypotensive effects on SHRs, improved iron solubility during gastrointestinal digestion, and a greater ability to transport iron across the monolayer of Caco-2 cells. Moreover, ferrous chelation did not alter the ACE inhibition mode or ability or the hypotensive effect of PMYGGGMV, but it decreased its antioxidant activity (*p* < 0.05). These findings show that PMYGGGMV has potential applications as an antioxidant, an antihypertensive agent, and in iron supplements. However, its specific effects on the Keap1-Nrf2-ARE system, in vivo antioxidant activity, and absorption mechanisms require further study.

## Figures and Tables

**Table 1:** Amino acid sequences, ACE inhibitory capacity, ferrous chelating activity, and in silico prediction of antioxidant activity, physicochemical properties, toxicity, and allergenicity of peptides identified in sweet almond globulin hydrolysates.

Peptide Sequence	AEAPKE	RSGGEEA	RFMTYS	PMYGGGMV	NDSAGS	RFMTYSSS	RSGGEEAA	SGGEEAAAGP
Mass (Da)	643.76	704.78	804.00	811.10	549.56	978.18	775.87	1001.16
Matched sequence in *Runus amygdalus* ^a^	G.AEAPKE.K	Q.RSGGEEA.A	G.RFMTYS.S	A.PMYGGGMV.T	A.NDSAGS.E	G.RFMTYSSS.L	G.RSGGEEAA.G	R.SGGEEAAAGP.G
SVMS ^b^	−0.52	−0.71	−0.71	0.77	−1.37	−0.99	−1.16	−0.14
Antihypertension prediction	Non-AHT	Non-AHT	Non-AHT	AHT	Non-AHT	Non-AHT	Non-AHT	Non-AHT
ACE inhibitory activity (IC_50_: μmol/L)	ND	ND	ND	121.16	ND	ND	ND	ND
Probability ^c^	0.10	0.07	0.34	0.56	0.16	0.19	0.07	0.17
Ferrous chelating capacity (mg/g)	18.22 ± 0.13 g	22.65 ± 1.09 f	0.19 ± 0.02 f	11.01 ± 0.43 h	7.36 ± 0.35 i	2.36 ± 0.17 j	3.27 ± 0.34 j	16.14 ± 0.43 g
Hydrophobic amino acid content (%)	33.33%	14.29%	33.33%	50.00%	16.67%	0.00%	25.00%	20.00%
Physicochemical properties								
Hydrophobicity	−0.32	−1.61	−0.22	0.19	−0.24	−0.23	−0.31	−0.19
Amphiphilicity	1.03	0.71	1.25	0.63	0.00	0.94	0.62	0.45
Hydrophilicity	1.33	1.26	−0.53	0.49	0.55	−0.33	1.04	0.71
Isoelectric point	4.54	4.54	9.10	5.88	3.80	9.10	4.54	4.54
Security ^d^								
Toxicity ^e^	−0.5	−0.5	−0.5	−0.5	−0.5	−0.5	−0.5	−0.5
Allergenicity	No	No	No	No	No	No	ND	ND

^a^ From the National Center for Biotechnology Information (NCBI); ^b^ SVMS (vector machine software score) and physicochemical properties were predicted in silico using the AHTPDB database; AHT: antihypertension; ^c^ the predicted probability for antioxidant activity using the database Peptide Ranker server; ^d^ the potential toxicity and allergenicity were predicted using the databases ToxinPred and AlgPred, respectively; ^e^ ‘−0.5’ means non-toxic peptides. ND: not measured. Different lowercase letters (f–j) in the same line mean significant differences (*p* < 0.05).

**Table 2:** Antioxidant activity of sweet almond globulin hydrolysates fraction D (SAGH-D) and antihypertensive peptides identified in SAGH-D at 100 μg/mL with glutathione as the comparison.

Samples	ABTS^+^ Scavenging Activity (%)		·OH Scavenging Activity (%)		Superoxide Radical Scavenging Ability (%)		Reducing Power	
	Before Gastrointestinal Digestion	After Gastrointestinal Digestion	Before Gastrointestinal Digestion	After Gastrointestinal Digestion	Before Gastrointestinal Digestion	After Gastrointestinal Digestion	Before Gastrointestinal Digestion	After Gastrointestinal Digestion
SAGH-D	84.28 ± 4.07 b	ND	87.63 ± 1.58 c	ND	37.85 ± 2.14 c	ND	0.325 ± 0.004 d	ND
PMYGGGMV	92.55 ± 0.76 a	87.67 ± 2.95 a	93.06 ± 2.55 b	96.53% ± 4.32% a	73.76 ± 3.58 b	69.38 ± 2.49 a	0.484 ± 0.012 b	0.476 ± 0.007 a
PMYGGGMV-Ferrous chelate	35.27 ± 2.45 d	ND	57.69 ± 0.07 d	ND	75.49 ± 4.09 b	ND	0.179 ± 0.003 e	ND
Glutathione	79.25 ± 1.67 b	ND	95.56 ± 1.38 a,b	ND	90.52 ± 4.97 a	ND	0.675 ± 0.019 a	ND

Different lowercase letters (a–e) in the same column mean significant differences; ND: not measured.

**Table 3:** Interactions between active sites of ACE or Keap1 with peptides identified from sweet almond globulin hydrolysates using a molecular docking simulation.

Peptides	T-Score	C-Score	Interaction Mode	ACE Residues and the Length of Hydrogen Bonds Formed Between ACE and the Ligand	T-Score	C-Score	Keap1 Residues and the Length of Hydrogen Bonds
PMYGGGMV	11.86	4.00	Hydrogen bond	Lys511: 3.17 Å; Tyr520: 2.64 Å; Tyr523: 2.98 Å; Arg522: 2.94 Å	8.51	4.00	Arg326: 2.79 Å; Val369: 3.11 Å; Val512: 3.15 Å; Val420: 2.98 Å; Val418: 3.14 Å; Arg415: 3.02 Å, 2.12 Å; Glu79: 3.19 Å
			Hydrophobic interaction	Asn277, Glu376, Lys454, Asp453, Phe457, Gln281, Thr282, Val379, Val380, His383, His387, His353, Glu411, Ala354, Val518, Glu384, Phe512, Ser355, Phe391, His410			

## Data Availability

The original contributions of this study are included in the article; further inquiries can be directed to the corresponding author.

## Abbreviations

The following abbreviations are used in this manuscript:

Keap1	Kelch-like ECH-Associated Protein 1
ACE	Angiotensin-I-Converting enzyme
Nrf2	Nuclear factor erythroid 2-related factor 2
SAGHs	Sweet almond globulin hydrolysates
SAGH-D	Sweet almond globulin hydrolysates subfraction D
PMYGGGMV	Pro-Met-Tyr-Gly-Gly-Gly-Met-Val
AEAPKE	Ala-Glu-Ala-Pro-Lys-Glu
RSGGEEA	Arg-Ser-Gly-Gly-Glu-Glu-Ala
NDSAGS	Asn-Asp-Ser-Ala-Gly-Ser
RFMTYS	Arg-Phe-Met-Thr-Tyr-Ser
RFMTYSSS	Arg-Phe-Met-Thr-Tyr-Ser-Ser-Ser
RSGGEEAA	Arg-Ser-Gly-Gly-Glu-Glu-Ala-Ala
SGGEEAAAGP	Ser-Gly-Gly-Glu-Glu-Ala-Ala-Ala-Gly-Pro
